# Acceptability and Feasibility of the Transfer of Face-to-Face Group Therapy to Online Group Chats in a Psychiatric Outpatient Setting During the COVID-19 Pandemic: Longitudinal Observational Study

**DOI:** 10.2196/27865

**Published:** 2021-07-23

**Authors:** Julia Scholl, Elisabeth Kohls, Frauke Görges, Marc Steinbrecher, Sabrina Baldofski, Markus Moessner, Christine Rummel-Kluge

**Affiliations:** 1 Department of Psychiatry and Psychotherapy Medical Faculty Leipzig University Leipzig Germany; 2 Department of Psychiatry and Psychotherapy Universitätsklinikum Leipzig Leipzig Germany; 3 Center for Psychotherapy Research Universitätsklinikum Heidelberg Heidelberg Germany

**Keywords:** online, group chats, COVID-19 pandemic, psychiatric outpatient setting, online interventions, e-mental health, COVID-19, pandemic, mental health, psychoeducation, online chat

## Abstract

**Background:**

At the height of the COVID-19 pandemic, several mental health care providers were obliged to shut down outpatient services, including group therapy and psychoeducational sessions. The lockdown in many countries is a serious threat to people’s mental well-being, especially for individuals with severe mental illnesses. Discontinued outpatient treatments and disruption of daily routines are considered to be risk factors for destabilization of patients with mental illness.

**Objective:**

The aim of this study was to evaluate the acceptability, usability, and feasibility of a group chat program to replace cancelled face-to-face group sessions in an outpatient psychiatric department.

**Methods:**

Participants (N=33) were recruited in the outpatient department of a large university medical center in Leipzig, Germany. Former face-to-face group participants were invited to take part in a therapist-guided group-chat for 4 weeks (8 sessions) and were asked to evaluate the program via self-administered standardized questionnaires at baseline (T0, preintervention), after every chat session (T1), and posttreatment (T2, after 4-6 weeks). The chat groups were specific to the following mental disorder diagnoses and based on the same therapeutic principles and techniques as the former face-to-face groups: anxiety, depression, obsessive-compulsive disorder, and adult attention-deficit/hyperactivity disorder (ADHD). Sociodemographic measures, attitudes toward the COVID-19 pandemic, depressive symptoms (Patient Health Questionnaire-9), quality of life (abbreviated World Health Organization Quality of Life assessment), treatment credibility/expectancy (Credibility Expectancy Questionnaire), and participants’ satisfaction (Client Satisfaction Questionnaire-8 [ZUF-8]) were measured.

**Results:**

Participants joined an average of 5 out of 8 offered chat sessions. Participation rates in the respective groups were highest in the ADHD group (8.6/11, 78%) and lowest in the anxiety group (3.7/9, 41%). The overall preintervention level of depressive symptoms was moderate and showed a slight, nonsignificant improvement at posttreatment (T0: mean 10.7, SD 5.5; T2: mean 10.2, SD 5.5). A similar result was observed regarding quality of life (T0: median 41.7-68.8; T2: median 50-70.3). Treatment credibility and expectancy scores were medium-high (T0: mean_credibility_ 18.1, SD 3.8; mean_expectancy_ 11.2, SD 5.1; T2: mean_credibility_ 17.1, SD 4.8; mean_expectancy_ 10.3, SD 5.8). Further, significant correlations were detected between posttreatment expectancy score and posttreatment PHQ-9 score (*r*=–0.41, *P*=.02), posttreatment physical quality of life (*r*=0.54, *P*=.001), and posttreatment psychological quality of life (*r*=0.53, *P*=.002). Overall, participants’ satisfaction with the program was very high, both after chat sessions and at posttreatment (ZUF-8: mean score 20.6, SD 1.0). Of all participants, a majority (27/31, 87%) rated the program as excellent/good and indicated they would recommend the group chat program to a friend in need of similar help (23/31, 74%).

**Conclusions:**

A therapist-guided group chat program to substitute outpatient group setting treatment during the COVID-19 lockdown was shown to be feasible, usable, and highly acceptable for participants. Web-based programs such as this one provide an easy-to-implement tool to successfully stabilize participants during a difficult time, such as the COVID-19 pandemic.

**Trial Registration:**

German Clinical Trials Register DRKS00021527; https://tinyurl.com/3btyxc2r

## Introduction

E-mental health and web-based interventions, as in, the provision of psychosocial or psychotherapeutic support through information and communication technologies, have emerged as an important area in psychotherapy service and research over the past decade [1-3]. The worldwide COVID-19 pandemic was an unexpected situation for most health care providers. In an attempt to reduce the risk of infections and due to governmental restrictions, many health care providers in afflicted countries drastically reduced outpatient treatment options for patients who were in need of outpatient face-to-face therapy, increasing the need to quickly implement other treatment options to support patients [4-6].

This need to find rapid solutions was further amplified by other problems caused by the pandemic and by government restrictions, such as fear and “lockdown loneliness,” which may increase the likelihood of deterioration and crises among individuals with impaired mental health [7,8]. Lockdown loneliness occurs because of required physical distancing, social isolation, and quarantine. For individuals with mental illnesses, it was necessary to discontinue many stabilizing factors during the pandemic, such as social contacts, group therapy, and face-to-face psychotherapy, leaving this group particularly vulnerable.

To address these challenges, an urgent need arose for treatment options that incorporated both therapeutic interventions and social interaction. Due to the extensive government restrictions, e-mental health interventions appeared to be the most promising approach.

Various web-based solutions had already been developed and thoroughly researched before the pandemic; however, these solutions encountered certain barriers when being implemented in routine care [9,10], especially due to the lack of acceptance by health care professionals themselves [11]. Because professionals were now forced to apply web-based technologies, it was predicted that the COVID-19 pandemic could be a turning point for e-mental health care [5].

During the pandemic lockdown, the face-to-face therapist-guided self-management groups in our psychiatric outpatient department were transformed into online group chats, similar to online support groups. These groups have been shown to be accessible and popular [12,13] and have potential to provide valuable support to individuals with depression as well as other common mental disorders [14]. Recent studies suggest that such support groups may improve mental health outcomes [15,16] and increase users’ sense of empowerment, self-esteem, and perceived quality of life [17].

The aim of this study is to investigate the acceptability, usability, feasibility, and user satisfaction of a therapist-guided online group-chat program as a substitute treatment during the COVID-19 pandemic. The mental state of the participants and their behavior regarding the use of support offers were assessed. The program was intended to stabilize patients’ mental condition during the high-risk situation of the COVID-19 pandemic.

## Methods

### Study Design

This longitudinal, observational prospective study evaluates a therapist-guided group chat intervention that was developed to replace face-to-face therapist-guided self-management group sessions during the lockdown phase of the COVID-19 pandemic. Groups were diagnosis-specific for participants with anxiety, depression, obsessive-compulsive disorder (OCD), and adult attention-deficit/hyperactivity disorder (ADHD), respectively. The study was approved by the ethics committee of the Medical Faculty of the University of Leipzig (133/20-ek, 03/2020) and is registered in the German Clinical Trials Register (DRKS00021527).

### Participants and Recruitment

Recruitment of participants started after the psychiatric outpatient department was forced to close and the face-to-face self-management group sessions could no longer take place due to governmental regulations in Germany during the COVID-19 pandemic in April and May 2020. Patients who were receiving treatment at the outpatient department and were part of the established diagnosis-specific psychoeducation and support groups were asked by their therapist whether they wanted to participate in online group chat sessions as a substitute treatment during the lockdown. The inclusion criteria were age 18 years or older, current treatment in the psychiatric outpatient department, former participation in group sessions, adequate understanding of the German language, sufficient sight and reading ability, internet access, and an e-mail address. There were no exclusion criteria. Participants were informed via telephone by their therapist about the group chat program and gave consent via telephone, followed by written informed consent. Paper-and-pencil questionnaires were sent as a small booklet via regular mail to all participants before the start of the intervention. After completing the intervention, participants sent back the questionnaires by prepaid return envelope. An e-mail reminder system to ensure the return of the completed assessments was implemented.

Through this process, 38 participants were included in the study, with an age range of 19-66 years and a mean age of 39.44 years (SD 12.28). The sample comprised an even gender distribution (female: 20/38, 53%; male: 18/38, 47%). The data of N=33 participants was analyzed (n=3 missing data; n=2 dropouts). Additionally, the posttreatment evaluation of 2 participants was filled in insufficiently, and therefore their data could not be analyzed.

### Chat Intervention

The intervention consisted of 8 chat sessions over a duration of 4 weeks. Sessions were scheduled twice a week at fixed times during April and May 2020 and lasted between 60 and 90 minutes. Sessions were moderated by psychotherapists from the outpatient department, who were experienced in administering group therapy and psychoeducation and who had also led the respective face-to-face group sessions before the lockdown. The website and general program for the chats already existed and was customized for our study. Participants needed to log in with a username and secure password to ensure data security. Screenshots of the interface can be seen in Multimedia Appendix 1. As the participants and therapists knew each other personally from the former face-to-face group sessions, chat sessions were (after agreement by all participants) not held anonymously. There were 4 chat groups by diagnosis: anxiety (n=9), depression (n=10), OCD (n=8), and ADHD (n=11). The same classification was used as in the preexisting groups. During chat sessions, participants and therapists were able to communicate by written messages and basic emojis. Therapists were asked to apply techniques equivalent to those they would apply in a face-to-face setting. There was a concrete and manualized psychoeducational theme schedule, although the therapists adapted the topics in the individual chats to participant suggestions or current problems. Additionally, the therapists moderated the exchanges between participants about their own experiences with their disorder as well as coping strategies for everyday life. The main objective of the chats was to stabilize and monitor participants’ mental situation and behavior. In cases of severe crisis or suicidal ideation, therapists were asked to perform the standard operating procedure from the outpatient department, adapted to the remote situation. It was not mandatory to participate in all 8 chat sessions.

### Measures

Outcomes were measured through self-report questionnaires (paper-and-pencil in a booklet) filled in by participants and sent back via regular mail. Assessments were conducted at baseline (T0, preintervention), after every chat session (T1), and posttreatment (T2, after 4-6 weeks).

#### Sociodemographic Measures

Participants were asked questions about basic sociodemographic characteristics, such as marital status, living situation, parenthood, employment status, and changes in employment due to the COVID-19 pandemic.

#### Satisfaction

After each chat session, the participants were asked to fill out an adapted 5-item version of the Client Satisfaction Questionnaire-8 (ZUF-8) [18,19] to measure their satisfaction with the sessions. A full 8-item version of the ZUF-8 was administered at the posttreatment evaluation. All items were measured on 4-point Likert scales. The total sum scores at the posttreatment evaluation ranged from 8 to 32, with higher scores indicating higher satisfaction. The reliability of the ZUF-8 is generally high, and its internal consistency is sufficient [18].

#### Depressive Symptoms

The Patient Health Questionnaire-9 (PHQ-9) [20-22] was administered at baseline evaluation and posttreatment to assess symptoms of depression. Symptoms were rated by 9 items on a 4-point Likert scale from 0, not at all, to 3, nearly every day. The total sum score ranges from 0 to 27, with higher scores indicating higher levels of depressive symptoms. The scores were categorized by levels of severity. Reliability and validity studies of the tool have indicated that it has sound psychometric properties. The internal consistency of the PHQ-9 has been shown to be high [20].

#### Quality of Life

The abbreviated World Health Organization Quality of Life assessment (WHOQOL-BREF) [23,24] was used to measure the participants’ quality of life at baseline and posttreatment. The 26 items on satisfaction with certain areas of life were rated on 5-point Likert scales from 1, not at all, to 5, extremely. The questions were divided into 4 different domains of quality of life: physical, psychological, social, and environmental. For each of those domains, an index was calculated, ranging from 0 to 100, with higher index scores representing higher quality of life. The WHOQOL-BREF has shown good discriminant validity, content validity, test-retest reliability, and internal consistency [23].

#### Treatment Expectancy and Credibility

To measure treatment credibility, participants completed the Credibility Expectancy Questionnaire (CEQ) [25] at baseline and posttreatment. The CEQ was translated using a back-translation procedure, and the wording was adapted slightly to the online format of the intervention. The questionnaire includes a credibility factor (3 items) and an expectancy factor (3 items). For the credibility factor, all items were measured on a rating scale of 1 to 9. Items 1 and 3 of the expectancy factor used an 11-point scale, from 0% to 100%, and item 2 also used a 1-9 rating scale. After transforming the percentage scales, the total sums of the scores for credibility and expectancy ranged from 3 to 27, with higher scores indicating a higher credibility/expectancy. The CEQ has demonstrated high test-retest reliability and adequate internal consistency [25].

#### Use of Other Support Services

In the posttreatment questionnaire, several items were included to inquire as to which supportive offers were used by the participants other than the group chat sessions (eg, offers by the psychiatric outpatient department, social media, or other internet-based support forums). The outpatient department additionally offered a hotline for patients with severe mental crises.

The primary outcome measure was defined as the participants’ satisfaction measured by the ZUF-8 at posttreatment as well as after every chat session. Secondary outcome measures were defined as the participants’ quality of life (WHOQOL-BREF), depressive symptoms (PHQ-9), and treatment credibility and expectancy (CEQ).

### Statistical Analysis

First, descriptive statistics were determined for socioeconomic variables, changes in employment due to the COVID-19 pandemic, symptoms of depression, quality of life, treatment expectancy/credibility, and participants’ satisfaction. Second, potential differences between depressive symptoms at baseline and posttreatment were tested with a *t* test for paired samples. For the different domains of quality of life (WHOQOL-BREF), Wilcoxon tests (nonparametric paired groups) were administered, because the tests for normal distribution (Shapiro-Wilks test) showed that the values of this outcome were not distributed normally. Finally, the correlations between treatment credibility/expectancy at baseline and posttreatment (credibility and expectancy factors of the CEQ) and posttreatment outcome (depressive symptoms of the PHQ-9 and quality of life of the WHOQOL-BREF) were analyzed using the Pearson correlation coefficient. All statistical testing was two-tailed at an level of .05. Analyses were performed using SPSS, version 25.0 (IBM Corporation).

## Results

### Sample Characteristics

A total of 33 participants were included in the final sample. Half of the 33 participants (n*=*16, 49%) were single, and 42% (n*=*14) were either married or in a relationship. A majority of the 33 participants (n*=*22, 67%) lived with other people (partner, children, roommates, etc). Approximately three-quarters were childless (24/33, 73%) and lived without children in their household (n*=*25, 76%). Only 39% (13/33) of the participants were currently employed, approximately 20% (6/33, 18%) were retired, and 15% (5/33) were unemployed. Due to the COVID-19 pandemic, more than half (9/16, 56%) of the working participants indicated that their work time had not changed, while one-quarter (4/16, 25%) indicated that their work time had decreased. Only 13% (2/16) lost their job due to the pandemic. A majority of 67% of the working participants (8/12) were working from home during the pandemic, and approximately one-quarter (3/12, 25%) were still working at their regular workplace.

### Participation

Participants joined an average of 5 of the scheduled 8 chat sessions (anxiety, mean 3.7; depression, mean 3.4; OCD, mean 5.3; ADHD, mean 6.2). The participation rates were highest in the ADHD group, with an average of 8.6 out of 11 participants (78%) joining the chat sessions. The lowest participation rates were detected in the anxiety group, with only 40% participation on average (3.7/9, 41%). In the depression group, approximately half of the participants joined on average (4.25/8, 53%), and in the OCD group, around 60% joined (5.3/8, 66%; see Table 1).

**Table 1:** Chat participation for each chat session per group; data are based on log-ins to the chatroom from n=36 participants.

Group	Participants in each chat, n							
	1 (mean 7)	2 (mean 4.5)	3 (mean (7)	4 (mean 6.7)	5 (mean 6.5)	6 (mean 4.3)	7 (mean 5)	8 (mean 5)
Depression (n=8, mean 4.3)	7	1	5	5	4	3	5	4
ADHD^a^ (n=11, mean 8.6)	11	9	9	9	9	8	7	7
Anxiety (n=9, mean 3.7)	2	4	7	5	5	4	2	4
OCD^b^ (n=8, mean 5.4)	8	4	7	3	8	2	6	5

^a^ADHD: adult attention-deficit/hyperactivity disorder.

^b^OCD: obsessive compulsive disorder.

### Satisfaction

In the postsession evaluations, a majority of participants (66%-87%) indicated that they thought the respective chat was excellent/good. Most of the participants received the support they wanted (59%-78%) and would recommend such a group chat program to a friend in need of similar help (70%-87%). The highest scores were achieved for the question of how the participants liked the therapeutic chat. A range of 81%-100% of participants indicated they thought it was excellent/good. The posttreatment evaluation showed a mean sum score of 20.6 (SD 1.0), which indicates a moderately high overall satisfaction with the program (see Table 2).

**Table 2:** Results of assessments at baseline (T0) and posttreatment (T2).

Variable			T0 (n=33)		T2 (n=31)		*P* value
**Depressive symptoms (PHQ-9^a^ score)**							
	0-4, n (%)	3 (9)		6 (19)			
	5-9, n (%)	10 (30)		7 (23)			
	10-14, n (%)	12 (36)		12 (39)			
	15-19, n (%)	6 (18)		5 (16)			
	20-27, n (%)	2 (6)		1 (3)			
	Sum score, mean (SD)	10.7 (5.5)		10.2 (6)		.93	
**Quality of life (WHOQOL-BREF^b^ score), median (IQR)**							
	Physical	55.4 (21.4)		57.1 (25.0)		.62	
	Psychological	41.7 (29.2)		50.0 (24.0)		.52	
	Social	58.3 (25.0)		58.3 (31.3)		.62	
	Environment	68.8 (20.3)		70.3 (14.8)		.14	
**Treatment credibility/expectancy (CEQ^c^ score), mean (SD)**							
	Credibility factor	18.1 (3.8)		17.1 (4.8)		N/A^d^	
	Expectancy factor	11.2 (5.1)		10.3 (5.8)		N/A	
Satisfaction (ZUF-8^e^ sum score), mean (SD)			N/A		20.6 (1.0)		N/A

^a^PHQ-9: Patient Health Questionnaire-9.

^b^WHOQOL-BREF: abbreviated World Health Organization Quality of Life assessment.

^c^CEQ: Credibility/Expectancy Questionnaire.

^d^N/A: not applicable.

^e^ZUF-8: Client Satisfaction Questionnaire-8.

### Depressive Symptoms and Quality of Life

In this sample, baseline and posttreatment evaluation of the PHQ-9 scores showed an average of moderate depressive symptoms (T0: mean 10.7, SD 5.5; T2: mean 10.2, SD 5.5). In a calculation of the respective categories, a majority of participants (T0: 20/33, 61%; T2: 18/31, 58%) showed moderate to severe symptoms (see Table 2). At the baseline evaluation of the WHOQOL-BREF for quality of life, participants reached the lowest index scores in psychological health (median 41.7, IQR 29.2) and the highest scores in environmental quality of life (median 68.8, IQR 20.3). Overall, scores showed a medium level of quality of life at baseline. In the posttreatment evaluation, the scores were slightly higher; again, psychological health received the lowest score (median 50.0, IQR 24.0) and environmental quality of life received the highest score (median 70.3, IQR 14.8; see Table 2). There was no statistically significant difference in depressive symptoms (PHQ-9 scores) before and after the intervention (*t*_30_=.89, *P*=.93). There was also no statistically significant difference in the scores for quality of life on the WHOQOL-BREF (*P*_phys_=.62; *P*_psych_=.52; *P*_soc_=.62; *P*_env_=.14) (see Table 2).

### Treatment Credibility and Expectancy

In the baseline evaluation, the participants achieved high credibility scores (mean 18.1, SD 3.8) but lower expectancy scores (mean 11.2, SD 5.1). In the posttreatment evaluation, both scores slightly decreased (credibility: mean 17.1, SD 4.8; expectancy: mean 10.3, SD 5.8) (see Table 2). A correlation analysis showed a significant negative correlation between the posttreatment expectancy factor of the CEQ and the posttreatment PHQ-9 sum score (*r*=–0.41, *P*=.02). In addition, a significant positive correlation was found between the posttreatment expectancy factor and the posttreatment physical quality of life score (*r*=0.54, *P*=.001), while the correlation between the posttreatment credibility score and the posttreatment physical quality of life score was not significant (*r*=0.35, *P*=.06). Lastly, significant correlations were shown between posttreatment psychological quality of life scores and expectancy factors at baseline as well as posttreatment (*r*=0.36, *P*=.046, and *r*=0.53, *P*=.002, respectively).

### Use of Other Support Services

In the posttreatment evaluation, use of other support services was assessed. In this evaluation, we could also include one of the insufficiently completed posttreatment questionnaires; therefore, the percentages were calculated with n=32. A majority of these 32 participants (n*=*19, 59%) indicated having had contact via telephone with their treating psychiatrist, while one-third (n=9, 28%) had contact with their treating psychotherapist. One-fifth of the participants used other offers, such as contact with the department’s social worker (6/32, 19%). Most of the participants (29/32, 91%) stated having had social contacts in the last few weeks, and approximately half (n=17, 53%) used social media on a daily basis. The most commonly used social media platform by the participants was the messaging platform WhatsApp, with 82% (14/17), followed by Facebook, with 47% (8/17). On average, social media platforms were used for 2.5 hours per day (mean 2.4, SD 1.7). Around half (n = 9, 52.9%) of the participants also used other social media, like other messaging services (Telegram, Threema, Discord), Twitter or YouTube (see Table 3). Other offers that provided support to participants were indicated by 21.9% (n=7, going for a walk, reading, having company, etc).

**Table 3:** Use of other support services by the study participants (n=32).

Question and answer choices					Value, n (%)
**Which offers by the psychiatric outpatient department helped you particularly well in the last few weeks?**					
	Telephone contact with treating psychiatrist			19 (59)	
	Telephone contact with treating psychotherapist			9 (28)	
	Contact in group chat			22 (69)	
	Others			6 (19)	
**Which other offers did you use in the last few weeks and thought to be especially helpful?**					
	Social contacts (eg, calls with friends and family)			29 (91)	
	**Social media**				
		Instagram users, n (%)	5 (29)		
		Time spent on Instagram (hours/day), mean (SD)	0.5 (0.3)		
		WhatsApp users, n (%)	14 (82)		
		Time spent on WhatsApp (hours/day), mean (SD)	0.9 (0.5)		
		Facebook users, n (%)	8 (47)		
		Time spent on Facebook (hours/day), mean (SD)	1.1 (0.8)		
		Other social media platform users, n (%)	9 (3)		
		Time spent on other platforms (hours/day), mean (SD)	1.7 (1.5)		
		Total social media users, n (%)	17 (53)		
		Total time on social media (hours/day), mean (SD)	2.4 (1.7)		
	Other online offers			5 (16)	
	Contact with treating therapist (outside psychiatric outpatient department)			5 (16)	
	Other contacts			7 (2)	

## Discussion

### Principal Findings

This study was conducted under the circumstances of the COVID-19 pandemic and its effects on individuals with mental health problems, which appear to be substantial [26]. During the lockdown in Germany in March and April 2020, psychiatric patients were confronted with the closure of outpatient departments and the suspension of support offers. Therefore, the program in this study was mainly designed to offer help and support to patients during this difficult time. The opportunity presented by this situation was used to investigate the acceptability and feasibility of the implementation of a therapist-guided group chat program in psychiatric outpatient care.

The transfer of face-to-face group therapies into online group chats in a psychiatric outpatient setting during the COVID-19 pandemic has been successful, as indicated by the results regarding the program feasibility, user satisfaction, and mental health status for all participants. First, the program was shown to be highly satisfactory for the participants, which indicates that the participants received the support that was intended to be given. Second, the program is feasible, as the depressive symptoms of the participants were successfully stabilized; these symptoms were initially at a moderate level and even showed a slight nonsignificant decrease at posttreatment. Similar results were observed regarding the participants’ perceived quality of life, which was stable at a medium level in all domains. Although the increase in quality of life scores was not statistically significant, the stabilization and lack of a significant decrease can be seen as successes, especially during the pandemic. The results are comparable to those of previous studies [27,28]. Because only approximately 40% of participants were employed at the time, all others lost their structured daily appointments at the psychiatric outpatient department, which are important in the treatment of mental illnesses. This program was seemingly able to support patients and stabilize their mental health.

The high satisfaction rates with the program after every chat and posttreatment show that the program is highly acceptable for patients experiencing severe mental illnesses. A majority of participants stated they would recommend the program to a friend in need of similar help, and the participants particularly liked the therapeutic chat. Compliance was good; on average, each participant joined 1 of 2 group chats per week, and more than half of the participants joined every chat.

Overall treatment credibility was medium-high; however, expectancy of improvement was rather low. Our results indicate that a high treatment expectancy correlates significantly with better outcome scores in posttreatment symptoms of depression and perceived quality of life, as patients with a higher treatment expectancy after the intervention had significantly lower PHQ-9 sum scores posttreatment. These findings are in line with previous research, which showed a strong association between treatment expectancy/credibility and outcome scores in different fields of research [29-32] as well as in mental health care [33].

In the last part of our evaluation, we observed that most participants used social contacts for support and found them to be the most helpful. Furthermore, a majority of participants were still in contact with their treating psychiatrist. In line with previous research [34], social media also proved to be an important part of participants’ support during this phase of isolation in our study. It can be concluded that additional internet-based treatment offers seem to be a feasible treatment option for most patients, especially during this pandemic.

### Limitations

It is important to emphasize that this is not a randomized controlled study and therefore should not be considered as a validation of the effectiveness of online group chat programs. However, preceding research has shown that online or web-based treatments are as effective as or even more effective than face-to-face interventions [35-43]. The aim of this study was to show that such programs are implementable if needed, and this is possible according to our results. Furthermore, online group chats seem to be highly acceptable for patients.

As this study was conducted during an exceptional situation for both patients and mental health care providers, this different environment must be considered when evaluating our results. Participants may have evaluated the program differently because they had no alternative therapy options for comparison at the time.

Participation notably differed between the diagnosis-specific groups, which may have been caused by different habits and behaviors that occur due to the respective illnesses. This finding may also have depended on how well the group members got along before the shift to online treatment.

### Conclusion

Online chat programs as a substitute for face-to-face group therapy sessions for patients with severe mental illnesses are feasible, usable, and can be implemented in clinical routine care under pandemic circumstances. They are also highly satisfactory and may have a stabilizing influence on patients’ symptom burden. Therefore, they can be considered as a substitute treatment for group therapy sessions during a pandemic, and they may be a helpful tool for future e-mental health offers in routine clinical care.

## Appendix

Multimedia Appendix 1 Screenshots of the chat program.

## Abbreviations

ADHD: attention-deficit/hyperactivity disorder
OCD: obsessive-compulsive disorder
PHQ-9: Patient Health Questionnaire-9
ZUF-8: Client Satisfaction Questionnaire-8
