# Supplementary figures and images for: Acceptability and Feasibility of the Transfer of Face-to-Face Group Therapy to Online Group Chats in a Psychiatric Outpatient Setting During the COVID-19 Pandemic: Longitudinal Observational Study
Source: JMIR Form Res. 2021 Jul 23;5(7):e27865. doi: 10.2196/27865 (PMC8315157; doi:10.2196/27865)

## Slide 1
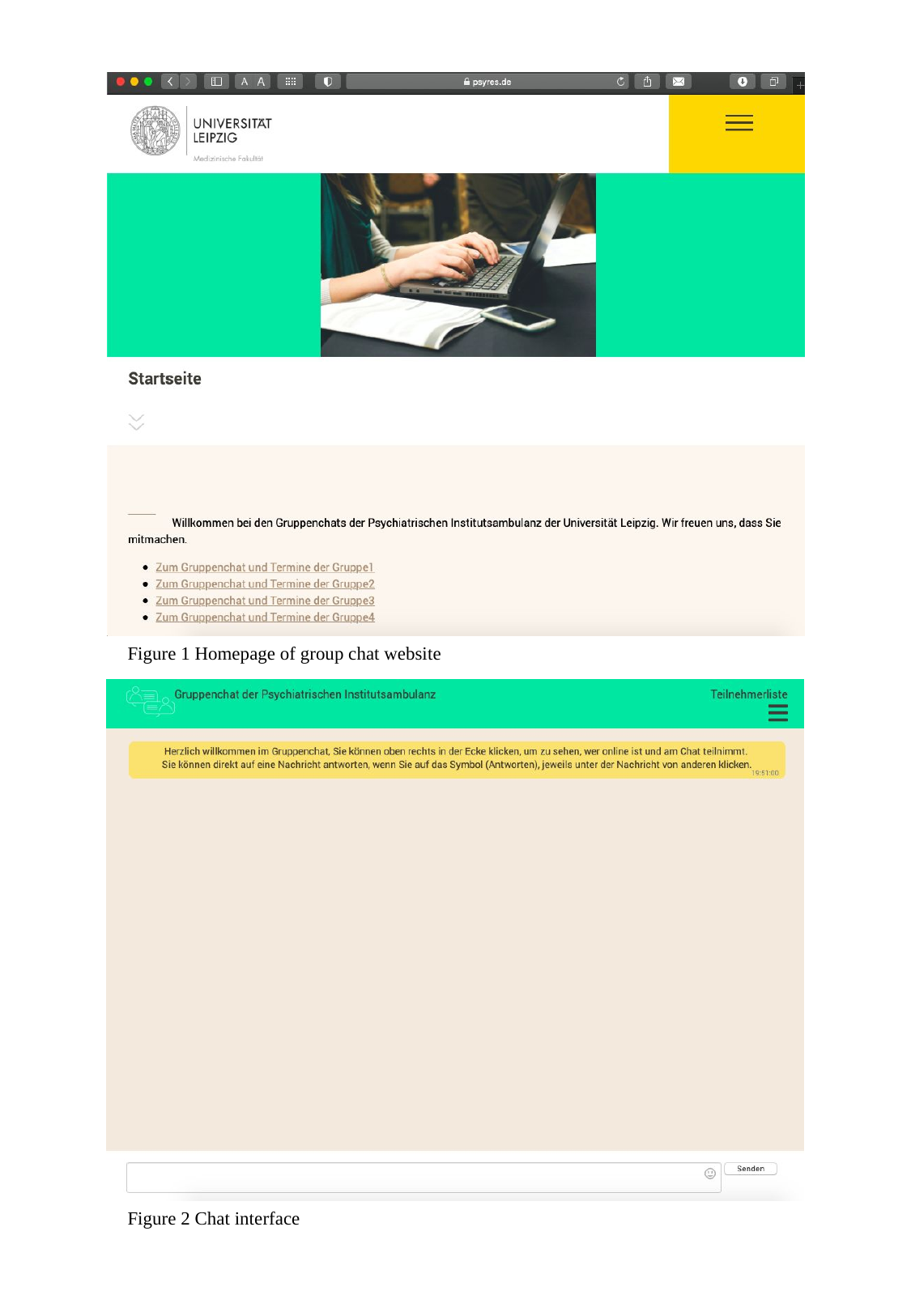

Figure 1 Homepage of group chat website
Figure 2 Chat interface

Supplement: Multimedia Appendix 1 [file formative_v5i7e27865_app1.pptx]
